# Physiological and Pathological Role of Alpha-synuclein in Parkinson’s Disease Through Iron Mediated Oxidative Stress; The Role of a Putative Iron-responsive Element

**DOI:** 10.3390/ijms10031226

**Published:** 2009-03-17

**Authors:** David Olivares, Xudong Huang, Lars Branden, Nigel H. Greig, Jack T. Rogers

**Affiliations:** 1 Neurochemistry Laboratory, Department of Psychiatry-Neuroscience, Massachusetts General Hospital (East), Harvard Medical School, CNY2, Building 149, Charlestown, MA 02129, USA; 2 Laboratory for High Throughput Biology, Yale University School of Medicine, West Haven, CT 06516-7381, USA; 3 Laboratory of Neuroscience, Intramural Research Program, National Institute on Aging, Baltimore, MD 21224, USA

**Keywords:** PD: Parkinson’s disease, AD: Alzheimer’s disease, α-syn: alpha-synuclein, PLD2: phospholipase D2, CNS: central nervous system, ER: endoplasmatic reticulum, PM: plasmatic membrane, LBs: Lewy bodies, LNs: Lewy neurites, GCIs: glial cytoplasmic inclusions, DLB: dementia with Lewy Bodies, DA: dopamine, DAT: dopamine transporter, NAC: non-amyloidogenic component, 5-UTR: 5’-untranslated region, IRE: iron responsive element, IRPs: interacting binding proteins, wt: wild-type, ROS: reactive oxygen species, GSH: reduced gluthatione, 6-OHDA: 6-hydroxydopamine, MPTP: 1-methyl 4-phenyl 1, 2, 3, 6 tetrapyridine, TfR: transferrin receptor, TH: tyrosine hydroxylase, nt: nucleotide(s), aa: amino acid(s)

## Abstract

Parkinson’s disease (PD) is the second most common progressive neurodegenerative disorder after Alzheimer’s disease (AD) and represents a large health burden to society. Genetic and oxidative risk factors have been proposed as possible causes, but their relative contribution remains unclear. Dysfunction of alpha-synuclein (α-syn) has been associated with PD due to its increased presence, together with iron, in Lewy bodies. Brain oxidative damage caused by iron may be partly mediated by α-syn oligomerization during PD pathology. Also, *α-syn* gene dosage can cause familial PD and inhibition of its gene expression by blocking translation via a newly identified Iron Responsive Element-like RNA sequence in its 5’-untranslated region may provide a new PD drug target.

## 1. Parkinson’s Disease: Clinical Profile, Pathophysiology and Treatments

### 1.1. Clinical profile

PD is a progressive neurodegenerative disorder that affects approximately 1% of the population beyond 65 years old [1]. A study of mortality among PD patients showed an *odds ratio* of 2.5 compared with age-matched subjects [2]. The diagnosis of PD continues to be based on presenting signs and symptoms. Dyskinesia is the most obvious clinical symptom and often starts in one extremity and worsens with stress, fatigue and cold. Bradykinesia is usually the most troublesome symptom. Patients refer to slowness in performing their daily activities. Rigidity of muscles on passive movement, including joints, is also a characteristic of PD [3].

### 1.2. Pathophysiology

The primary brain abnormality found in all patients is a degeneration of nigrostratial dopaminergic neurons in the *substantia nigra*, which leads to the depletion of dopamine (DA) with consequent loss of neuronal systems responsible of motor functions, and the formation of intracytoplasmic inclusions called Lewy bodies (LBs) in remaining neurons [4].

It is thought that the cause of idiopathic PD may be an interaction of environmental and genetic factors [4]. More typically, PD is sporadic when there is no family history of disease although a study has suggested a significant contribution of heredability to the development of late-onset PD [5]. In pedigrees with autosomal-recessive early-onset parkinsonism, a wide variety of mutations to the *parkin* gene (Park-2 gene) were found in families in which at least one member developed the symptoms [6]. Also, a number of hereditable genetic autosomal-dominant mutations have been found in the *α-syn* gene (also known as *SNCA*), besides other genes, as rare cause of PD, helping in understanding the disease [5]. α-syn has received much attention because it is the major component of LBs. In addition, α-syn pathologies accumulate throughout the central nervous system (CNS) in areas that also undergo progressive neurodegeneration, leading to dementia and other behavioural impairments as well as parkinsonism [7].

### 1.3. Treatments (see Table 1)

Although significant advances have been made in understanding the pathophysiology of this disease, there is no curative treatment and only symptoms can be controlled. The management of PD is designed to improve the patient’s quality of life. Symptomatic therapy is based totally on the requirements of the individual patient and must be re-evaluated as the condition evolves. Neuroprotective therapy is currently unavailable, in spite of the initial promising data from the DATATOP study performed with the monoamino oxidase (MAO) inhibitor selegiline, definitive neuroprotective action has yet to be demonstrated and its actions can be off set by its side effects that may include nausea, dizziness, insomnia and cognitive impairment. Actually, the American Academy of Neurology suggests that there is currently insufficient evidence to recommend selegiline as a neuroprotective treatment [8].

l-Dopa is presently the most efficacious treatment for PD [9]. l-dopa is converted into DA within the nigrostratial neurons by the enzyme aromatic l-amino acid decaboxylase. This enzyme is a rate-limiting in DA synthesis in PD, but not in healthy individuals, and pyridoxal phosphate (vitamin B6) is a required cofactor for the decarboxylation which may be administered together with l-dopa as pyridoxine. Conversion of l-dopa to DA likewise occurs systemically, outside the brain, in peripheral tissues, and thereby may induce adverse effects. It is hence standard clinical practice to co-administer a peripheral DOPA decarboxylase inhibitor that is restricted from entering brain, such as carbidopa or benserazide, and often a catechol-*O*-methyl transferase (COMT) inhibitor, to prevent peripheral tissue synthesis of DA. Essentially, all patients require l-dopa at some stage of the disease. However, careful l-dopa titration is essential since it may induce dyskinesias and other l-dopa side effects. The most common is the “wearing-off” phenomenon or shortening of sustainable pharmacological action. This occurs when the symptoms of PD, attenuated by the treatment, become more intense before the next dose. The response usually is an increase of l-dopa dose frequency and addition of alternative therapies, such as the COMT inhibitor entacapone, DA agonists, amantadine or selegiline [10] (see below). The major inconvenience of l-dopa is dyskinesias or involuntary movements related to the drug. The addition of amantadine usually attenuates dyskinesias [11]. Although l-dopa is associated with motor complications, it must be acknowledged that survival has been considerably prolonged in PD since its introduction. Moreover, as in healthy animals, individuals misdiagnosed with PD and exposed to long-term l-dopa treatment did not show signs of parkinsonism [12,13].

In specific clinical situations, lower potency drugs are used. As listed in Table 1, anticholinergics (benztropine, biperiden, diphenhydramine, ethopropazine, orphenadrine, procyclidine and trihexyphenidyl) provide mild symptomatic treatment and may be beneficial to treat tremors [14]. Unfortunately, many patients experience cognitive change following anticholinergics, and therefore they are generally restricted to younger patients. Amantadine, a *N*-methyl-d-aspartate receptor antagonist, also provides mild symptomatic benefit in early stages of disease. This agent is relatively inexpensive, with a low incidence of adverse effects but is also associated with confusion in older patients [10]. DA agonists (mainly bromocriptine, pramipexole, ropinerole and pergolide) directly stimulate DA receptors and do not need to be metabolized into an active drug. They may hence have potential advantages over l-dopa by having a longer half-life, a longer duration of symptomatic action and, most importantly, DA agonists are rarely associated with motor fluctuations and dyskinesias [15]. Nevertheless, patients may succumb to other side effects, such as hallucinations, hypotension, anxiety, depression, bladder dysfunction, insomnia, edema and cognitive impairment.

Several clinical trials have shown a decrease of dystonia and dyskinesia, and a similar clinical benefit when patients with early stage PD were treated with the DA receptor agonist bromocriptine, or l-dopa alone [16]. A similar result was obtained by using ropinirole plus l-dopa, if required [17]. These data support the use of DA agonists in early PD together smaller doses of l-dopa to minimize complications related to the latter [15]. In addition, several *in vitro* and animal studies might indicate a role for neuroprotection for DA agonists [18–21], although clinical benefit in humans is to prove. Other DA agonists, apomorphine, lisuride and carbegoline are also available, however, the choice of one them taking into account their effectiveness and/or non-motor symptoms remains unclear.

In addition, COMT is a selective and widely distributed enzyme involved in the catabolism of l-dopa. Tolcapone and entacapone are selective and potent COMT inhibitors that slow the metabolism of l-dopa, thus prolonging its effects [22]. Tolcapone has been shown to be an effective adjunct in the treatment of PD in Phase II and III clinical trials, improving motor fluctuations and reducing l-dopa requirements. Rare reports of severe hepatotoxicity, however, have limited tolcapone implementation in the treatment of PD [23].

The currently available therapies for PD, as in Table 1, are symptomatic and become less effective over time. Therefore, in order to identify potential neuroprotective agents available for testing, the Committee to Identify Neuroprotective Agents in Parkinson’s (CINAPS), supported by the National Institute of Neurologic Disorders and Stroke (NINDS), conducted a systematic assessment of presently available pharmacologic agents. From a list of 59 potential neuroprotective agents, the Committee identified 12 agents that are currently available and should be considered priority agents for further investigation in PD [24] (see Table 2, which includes neuroprective agents).

## 2. alpha-Synuclein: Relationship with PD. Biochemical and Biological Properties

### 2.1. Relationship with PD

As mentioned above, PD neurodegeneration is accompanied by the presence of LBs and Lewy neuritic inclusions (LNs) in surviving dopaminergic neurons [25], in which the main component derives from fibrillar aggregates of α-syn [26,27], although α-syn inclusions are also found in non-dopaminergic neurons (called neuronal cytoplasmic inclusions), in glial cells (glial cytoplasmic inclusions, GCIs) and as axonal spheroids [28].

The main evidence of the relevant role of α-syn in PD came from the discovery of three point mutations in the α*-syn* gene that can cause hereditable early-onset PD [29] in rare pedigrees. These mutations are A53T (change Ala in position 53 to Thr) [30], A30P (change Ala in position 30 to Pro) [31] and E46K (change Glu in position 46 to Lys) [32]. All three occur within the *N*-terminal side of the protein and are able to accelerate the α-syn *oligomeric* aggregation process and protofibril formation [33] faster than wild-type (wt) α-syn. Therefore, the insoluble *fibrillization* rate was also higher (with the exception of the A30P mutation) than in wt variant [34,35], leading to pathologic inclusions, such as LBs and LNs.

Interestingly, several clinical features may be distinguished among A53T carriers *vs.* idiopathic PD patients, such as a slightly earlier onset, a faster disease progression, a lower tremor prevalence, and dementia [36]. By contrast, patients with the A30P mutation resemble idiopathic PD. The E46K patients exhibit dementia with Lewy Bodies (DLB) and hallucinations, as well as parkinsonism. This mutation, which substitutes a dicarboxylic amino acid, glutamic acid, with a basic amino acid such as lysine in a much conserved area of the protein, is likely to produce a severe disturbance of protein function [32]. On the other hand, genetic polymorphisms in the α*-syn* gene appear to confer risk for sporadic PD [37]. In fact, in the Japanese population there are several polymorphisms in the intron 1 that may be associated with PD [38]. Furthermore, duplication or triplication of the α*-syn* gene has been reported in familial forms of PD [39], and could implicate gene dosage effects in the PD pathogenesis [40].

In agreement with genetic and clinical findings, transgenic animal models have revealed an association between α-syn and the disease. In *Drosophila,* for example, when wt- α-syn, A30P or A53T mutants are overexpressed, a motor dysfunction, selective loss of DA neurons and presence of filamentous intraneuronal inclusions that contain α-syn occur [41]. In contrast, α-syn knock-out mice possess an abnormal activity of DA neurons with reduced levels of DA detected in the striatum. This implies that the protein may play a role in the regulation of neurotransmitter release [42].

The importance of the link between α-syn and PD, together with the discovery of detectable levels of α-syn in CSF and in human plasma, suggesting that α-syn is released from affected dopaminergic neurons [43], indicates that α-syn could serve as a marker for early diagnosis of PD. In this regard, an Enzyme-Linked Immuno Sorbent Assay (ELISA) based method has recently been developed to detect oligomeric α-syn in CSF and plasma and could serve as a diagnostic tool for PD and related diseases [44].

### 2.2. Biochemical and biological properties

α-Syn belongs to the synuclein family, which includes β-syn and γ-syn [45]. α-syn and β-syn are predominantly expressed in brain at presynaptic terminals, particularly in the neocortex, hippocampus, striatum, thalamus and cerebellum [46,47]. γ-syn is highly expressed in several areas in the brain, particularly in the *substantia nigra,* and has been found to be overexpressed in some breast and ovarian tumors [48]. α-syn homologues have been found in several mammals, but not in lower organisms such as *Escherichia coli,* yeasts, *Drosophila* or *Caenorhabditis elegans.*

α-Syn is a small protein (14 kDa) composed of 140 aminoacids (aa). It is a soluble, acidic, resistant to high temperatures and natively unfolded protein with an extended structure that is mainly composed of random coils [49]. However, it acquires secondary structure elements upon interaction with ligands and proteins, adopting a partially folded conformation [50]. A report suggests that unfolded states play a functional role in vesicle fusion in all eukaryotics, bringing membrane surfaces to facilitate fusion, and, after binding, an ordered structure is acquired [51].

**Figure 1.**
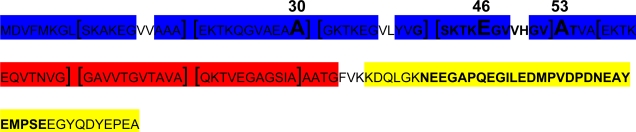
Alpha-synuclein’s sequence and domains. Blue highlighted: four α-helices responsible for protein-membrane interactions. Red highlighted: NAC or non-Aβ (amyloidogenic) component of α-syn, responsible of protein-protein interactions. Yellow highlighted: the unstructured C-terminal domain. Exons that undergo alternative splicing are indicated in bold: exon 3 from codon 41 to 54 and exon 5 from codon 103 to 130. Mutations A30P, E46K and A53T are in bold and enhanced. The seven 11 aa repeats are shown between the square brackets.

The α-syn sequence can be subdivided in three domains (Figure 1). The highly conserved N-terminal domain (residues 1–65) is unordered in solution and includes seven copies of an unusual 11 aa repeat that displays variations of a KTKEGV consensus sequence that may shift to an α-helical structure under certain conditions [52,53]. α-syn can interact with synthetic phospholipid vesicles through this domain [54], suggesting that the protein may be membrane-associated [55], becoming α-helix conformation after binding [56].

The A53T and A30P mutations do not affect the overall structure of α-syn, which remains unfolded, but diminish hydrophobicity of the N-terminal domain; thereby somewhat reducing its ability to form α-helices. The predisposition to form β-sheet structures is enhanced [50], which renders it more prone to aggregation. Whereas A53T does not affect vesicle binding, A30P is characterized by a decrease in lipid binding [33].

The central hydrophobic domain of α-syn (residues 66–95) is known as NAC. This peptide portion has been implicated in AD pathogenesis and is the second major component of brain AD amyloid plaques [57]. It is responsible for protein-protein interactions and confers on α-syn the ability to undergo a conformational change from a random coil to an aggregation-prone β-sheet structure [58] leading to the formation of amyloid-like fibrils [59]. An *in vitro* study demonstrated that aged NAC, dissolved in solution for seven days, is more toxic than fresh NAC, suggesting that cytotoxicity depends on prior aggregation [60]. The NAC region also contains a phosphorylation site on Ser-87.

The acidic Glu-rich C-terminal domain (residues 96–140) has no recognized structure and a strong negative charge [52]. Several phosphorylation sites have been identified within this region, on Tyr-125, −133 and −136, and Ser−129 [61]. Its acidic domain (aa 125–140) appears to be critical for the chaperone activity of α-syn [62]. Chaperones are proteins that prevent irreversible protein aggregation and facilitate the correct folding of proteins through binding *in vivo*. α-syn shares a 40% homology with a chaperone called 14-3-3 [63], suggesting that both proteins may share the same function. Chaperone 14-3-3 is particularly abundant in brain and can prevent apoptosis by binding with the pro-apoptotic protein, BAD [64]. α-Syn selectively interacts with 14-3-3 in *substantia nigra,* where it accumulates in LBs, leading to a decrease in available levels of 14-3-3 to inhibit apoptosis [65]. Both α-syn and 14-3-3 interact with tyrosine hydroxylase (TH), the rate-limiting enzyme in catecholamine synthesis that is responsible for catalyzing the conversion of the amino acid l-tyrosine to l-dopa. TH activity is stimulated by 14-3-3 and inhibited by α-syn [66].

#### 2.2.1. Posttranslational modifications

All posttranslational modifications of proteins result in a change of protein size, structure or charge, leading to alterations of their original properties [67]. With regard to α-syn, there are several modifications:

#### 2.2.1.1. Phosphorylation

Ser-129 was established as a major phosphorylation site, while another was identified at Ser-87. Casein kinases, CK1 and CK2, were found to be responsible for α-syn phosphorylation at Ser-129 and, probably, at Ser-87. Both are localized in the synaptosome and also phosphorylate other synaptic vesicle proteins [68]. In addition, the G-protein-coupled receptor kinases can phosphorylate α-syn [69].

It has been determined that more than 90% of insoluble α-syn in LBs is phosphorylated. By contrast, phosphorylation involves only about 4% of normal α-syn, suggesting that phosphorylation is a relevant pathogenic event [70,71]. In this regard, phosphorylation at Ser-129 increases fibril formation [71].

Interestingly, cotransfection experiments of α-syn and synphilin-1, a protein that interacts with α-syn, yielded cytoplasmic inclusions that were similar to LBs. Subsequent coexpression of S129A α-syn (that is unable to be phosphorylated at Ser-129), synphilin-1 and parkin (a ubiquitin ligase responsible for α-syn and synphilin-1 ubiquitination), showed an important decrease in cytoplasmic inclusions [72], indicating that phosphorylation at Ser-129 enhances the formation of inclusion bodies and is a necessary step in the development of LBs. Transgenic mouse models that overexpress α-syn have shown a neurodegeneration that is accompanied by phosphorylation at Ser-129, caspase 9 activation and apoptosis [73].

##### 2.2.1.2. Nitration

Nitration has been proposed as one of the oxidative mechanisms responsible of the formation of α-syn oligomers, through di-tyrosine crosslinking [74]. Soluble nitrated α-syn is not efficiently processed by proteases, leading to partial folding, accumulation and fibril formation [75]. Consequently, nitrated α-syn has been found in LBs deriving from brains with PD [76]. The primary sequence of α-syn contains four sites for potential nitration: Tyr-39, −125, −133 and −136, all of which have been found nitrated in LBs [77].

##### 2.2.1.3. Ubiquitination

Protein modification by ubiquitin is one of the main mechanisms of protein targeting for proteasome degradation. Specifically regarding α-syn, the specific substrate for ubiquitination by parkin is the *O*-glycosylated α-syn form, with glycosylation potentially occurring at Ser-129 [78]. α-Syn ubiquitination occurs *in vivo* at Lys-6, −10 and −12. Nevertheless, it remains unclear whether monomeric α-syn requires ubiquitination, since α-syn is a natively unfolded protein and, therefore, it may not require ubiquitination and unfolding. Instead, it could enter the 20S proteasome directly [67]. In contrast, ubiquitin moieties are present in LBs associated with α-syn aggregates [79]. In addition, only high molecular weight α-syn fibrils, and not the monomeric protein, are substrates for oligoubiquitination in sporadic LB diseases [80]. It thus appears that ubiquitination of α-syn is a pathologic event associated with LB formation. Moreover, α-syn and parkin colocalize together in LBs, and a report suggests that α-syn aggregation could precede ubiquitination [81].

Several mutations associated to early-onset PD have been described in genes related to ubiquitination, for example, within the ubiquitin C-terminal hydrolase gene [82], thereby highlighting the importance of the misfunction of this mechanism in parkinsonism.

Protein degradation is tightly regulated in eukaryotic cells, and unfolded proteins, like α-syn, have a reduced lifetime and undergo a fast turnover. In many cases, this turnover is mediated by sequences rich in Pro, Glu, Ser and Thr, which target the protein for proteolysis [83], and are often present within a highly charged and unstructured region, preferentially in the C-terminal domain.

#### 2.2.2. α-Synuclein alternative splicing

Alternative splicing is a mechanism to support the generation of multiple mRNAs from a single transcript. Each alternatively spliced transcript contains significant changes in protein secondary structure that may cause functional alterations, and shifts the protein isoform ratio. There are two types of transcripts from pre-mRNA alternative splicing: C-terminal truncated proteins generated by reading frame changes that result in the introduction of a premature stop codon, and in-frame deletions with exon loss without a frame shift. With regard to α-syn, the 140 aa isoform is the whole protein and also the major transcript, whereas alternative splicing of exons 3 or 5 gives rise to 126 or 112 aa isoforms, respectively, both from in-frame deletions [67]. In addition, a 98 aa isoform of α-syn, which lacks exons 3 and 5, has been recently reported [84]. The splice-out of exon 3 results in the interruption of the helical protein-membrane interacting domain by deleting most of helix 3 and part of helix 4, and thereby impairing the aggregation-prone interaction with membranes [85]. Interestingly, the E46K and A53T mutations are sited in exon 3.

α-Syn 112 lacks exon 5 (aa 103–130) located on the C-terminal half of the protein. It shows an enhanced tendency to aggregate and fibrillize [86], in spite of the lack of the main phosphorylation site located at Ser-129 (although maintaining the phosphorylation site at Ser-87). Thus, either phosphorylation or structural alterations are responsible for aggregation, but other explanations arise, such as the lack of the proteolysis signal sequence at the C-terminus, leading to accumulation and aggregation. In addition, it has been proposed that the C-terminus may act as an intramolecular chaperone, preventing α-syn fibrillization [62].

A differential mRNA expression study [87] revealed different α-syn expression levels when comparing patients with DLB to controls. α-syn 140 expression levels were diminished as result of neuronal loss and an important 5-fold decrease of α-syn 126, together with a two-fold increase of α-syn 112 and α-syn 98 were described [84], suggesting an involvement of these latter isoforms in DLB pathologies that likely related to LBs formation. The decrease in α-syn 126 expression probably unbalances the ratio among the three isoforms towards the more prone to aggregate 112 aa isoform, albeit that no pathological processes related to α-syn 126 have yet been reported.

#### 2.2.3. Vesicle trafficking regulation

α-syn has the ability to bind phospholipid vesicles through its *N*-terminal domain, with four amphipathic α-helices that are typical of lipid-binding proteins. It is found in pre-synaptic termini, in equilibrium between free and membrane-bound states [88], with approximately 15% of α-syn being membrane-bound [55]. This led to the hypothesis that α-syn might regulate vesicular release and/or turnover and other synaptic functions within the CNS [59]. Expression profiling in transgenic flies revealed that expression of lipid and membrane transport genes were associated with α-syn expression [89]. Furthermore, overexpression of α-syn was accompanied by noticeable changes in membrane fluidity and in fatty acid uptake and metabolism [90].

An analysis of a yeast PD model revealed that the earliest defects following α-syn overexpression were an inhibition of the endoplasmatic reticulum (ER) to Golgi vesicular trafficking and an impairment of the ER-associated degradation [91]. In PC12 cells, α-syn regulates catecholamine release from synaptic vesicles, and its overexpression inhibits the vesicle priming process after secretory vesicle trafficking to docking sites [92]. Finally, α-syn appears also to be involved in the regulation of certain enzymes, transporters and neurotransmitter vesicles [93].

#### 2.2.4. Interaction with other proteins

α-Syn acts as a specific inhibitor of phospholipase D2 (PLD2) [94], which hydrolyzes phosphatidylcholine to phosphatidic acid (PA) [95]. Activation of PLD2 and generation of PA elicits a wide array of cell responses, including Ca^2+^ mobilization, secretion, superoxide production, endocytosis, exocytosis, vesicle trafficking, recycling of membrane receptors, transport to Golgi, rearrangements of cytoskeleton, mitogenesis and cell survival. PA can serve as a protein attachment site, altering membrane curvature and vesicle fusion [94].

Phosphorylation of α-syn by G-protein coupled receptor kinases results in a significant reduction in the α-syn affinity for phospholipids and a decrease in its binding with PLD2. Once detached from the plasmatic membrane (PM), α-syn is able to release any membrane-bound PLD2, allowing potential hydrolysis of phosphatidilcholine, the major lipidic component of cell membranes, to potentially increase membrane permeability [4].

#### 2.2.5. Chaperone activity

As discussed, α-syn has chaperone functions, assisting in the folding and unfolding of many synaptic proteins. In addition, transgenic mice with the cysteine-string protein-α (CSPα, a synaptic vesicle protein that acts as a co-chaperone) gene deleted have a phenotype of neurodegeneration. However, transgenic expression of α-syn prevented the development of this pathological sign. Thus, α-syn appears to complement the chaperone activity of CSPα [96].

## 3. Pathogenic Mechanisms of Alpha-Synuclein. Factors Affecting Fibrillization. Role of Iron in Oxidative Stress

### 3.1. Conformational states of α-synuclein

The structural disposition of α-syn can show under a number of different conformations:

1. The intrinsic unfolded state under physiologic conditions, both *in vitro* and *in vivo.*
2. The pre-molten globule state, a compact but incompletely folded state of proteins that contains most of the secondary structure but lacks tertiary interactions [97], and is predominant under conditions such as low pH, high temperature, several metal ions [98], several salts [98], and several common pesticides/herbicides [99]. It is stabilized as result of spontaneous oligomerization both *in vivo* and *in vitro* [100]. It is thought that the negative electrostatical potential and locally low pH in the vicinity of the membrane surface induces protein conformation to molten globules. Early stages of fibrillization involve the partial folding of α-syn into the highly fibrillization-prone pre-molten globule conformation, which represents a key intermediate along the fibrillization pathway [101].
3. The α-helical membrane-bound form in the *N*-terminal fragment, while the glutamate-rich *C*-terminal region remains unstructured.
4. The β-sheet state: it has been observed that under certain conditions α-syn acquires a β-pleated sheet, which is very prone to form amorphous aggregates [102].
5. Dimers: α-syn is able to form morphologically distinct oligomers, for example under high temperature [100], where dimers are formed first, and aggregates. The formation of oxidative dimers and high-order oligomers with covalent di-tyrosine cross-links under conditions of oxidative stress has also been reported [74].
6. Oligomers: nitrated α-syn assembles into spherical oligomers [100]. Incubation of α-syn with several metals gave rise to different classes of oligomers: Cu^2+^, Fe^3+^ and Ni^2+^ yielded 0.8–4 nm spherical particles, similar to those formed by incubation of α-syn alone; Mg^2+^, Cd^2+^ and Zn^2+^ yielded larger (5–8 nm) spherical oligomers; and Co^2+^ and Ca^2+^ produced ring oligomers with diameters between 70–90 nm for the former and 22–30 nm in the case of the latter [103]. It has been observed that the earliest form of α-syn protofibrils appeared to be mainly spherical [35]. The incubation of spherical α-syn oligomers with brain-derived membranes has been shown to produce pore-like ring-type protofibrils [103], which may disturb ionic gradients in cells. This conjecture was supported by showing that α-syn oligomers (and not monomeric or filamentous α-syn) enhanced the membrane permeability for Ca^2+^, an important subcellular messenger in liposomes [104]. The role for membrane permeabilization by α-syn *in vivo* is not clear and a large number of pores could lead to cell lysis, but even a subtle ionic disturbance could lead to neuronal dysfunction, death and degeneration.
7. Insoluble aggregates: finally, α-syn have been shown to assemble into large, insoluble aggregates with two distinct morphologies (amorphous aggregates and fibrils), with a high amount of β-sheet structure.

Because of this number of possible structural conformations, it seems reasonable to suggest that α-syn is potentially prone to misfold [105]. Fibrillization is a nucleation polymerization process, in which there is an initial lag phase, during which nuclei are formed, followed by the exponential growth of the fibrils, and an equilibrium phase between the protein in solution and the protein in fibrillar form [49]. The fibrillization rate increases with higher α-syn concentration, low pH, high temperature, oxidative stress-inducing compounds such as DA [106], the dopaminergic neurotoxin 1-methyl-4-phenyl-1,2,3,6-tetrapyridine (MPTP) [107], lipids [54] and pesticides [99]. The insoluble aggregates might represent the major building blocks of synucleinopathies-related inclusions, as LBs, LNs, GCIs and axonal spheroids, all of them observed in PD and related pathologies, as well as in other CNS disorders, including AD.

### 3.2. Factors affecting α-syn fibrillization

As stated above, a number of environmental and genetic factors can induce partial folding of α-syn, and therefore these same factors are able to accelerate the fibrillization process. Other aggregation-prone factors are:

1. Oxidation: the exposure of α-syn to oxidative agents induces the formation of high-order oligomers [74], and both the familial Parkininsonian A30P and A53T mutants have shown an even higher rate of self-assembly [108], providing support for the hypothesis that an impairment of cellular antioxidative mechanisms and/or overproduction of reactive oxygen species (ROS) may cause the initiation and progression of neurodegenerative synucleinopathies [109]. However, all amino acids are susceptible to oxidation [110], methionine being one of the easiest to undergo oxidation to form methionine sulfoxide (MetO). Thus, contrary to expectations, under mild oxidative conditions, when all four Met in α-syn are oxidized to MetO, the oxidized α-syn was found to be more unfolded than the non-oxidized form and less prone to oligomerize and fibrillate. Indeed, it even proved able to inhibit the fibrillization of non-modified α-syn [111].
  DA metabolism in nigrostratial neurons can produce ROS and contribute to lipid peroxidation, DNA damage, impairment of mitochondrial function, depletion of reduced glutathione (GSH), and, finally, cell death [112]. Over-expression of α-syn, and especially its mutant forms, enhances the vulnerability of neurons to DA-induced cell death through massive generation of ROS [113]. It has been proved that conjugation of DA with α-syn impedes the protofibril-to-fiber transition and, therefore, potentially more cytotoxic protofibrils may accumulate [114]. Transfection of wt- α-syn, A30P or A53T mutants has been reported to trigger apoptosis of cultured dopaminergic neurons, whereas there was an increase in survival of non-dopaminergic neurons [65]. Inhibition of DA synthesis by blocking TH activity prevented α-syn induced apoptosis.
  Damage caused by DA species is mediated by DA auto-oxidation catalyzed by free metals (especially Fe) [115], yielding 6-hydroxydopamine (6-OHDA) or through enzymatic deamination by MAOs to form toxic DA metabolites and hydrogen peroxide (H_2_O_2_) [116]. Levels of MAO-B appear to be highest in the *substantia nigra*. H_2_O_2_ produced as a by-product of DA oxidation and as normal oxygen reduction by MAOs is highly permeable and cannot be converted to water by GSH peroxidase due the low level of available GSH as a reducer [117], allowing H_2_O_2_ to potentially diffuses out of dopaminergic neurons and damage the neighbouring neurons. Under normal conditions, ROS are kept under control by an efficient antioxidant cascade. This includes the cytosolic copper-zinc superoxide dismutase and the mitochondrial manganese superoxide dismutase, which convert superoxide to oxygen and H_2_O_2_. The latter, in turn, is removed by catalases and peroxidases. These enzymes are key to scavenge ROS generated by oxidative insults. For example, in a transgenic murine model that overexpressed Cu/Zn superoxide dismutase or GSH peroxidase and was treated with pesticide paraquat, which causes a PD-like profile, the transgenic animals did not show alterations as reductions in locomotor activity, levels of striatal DA and metabolites, or dopaminergic neurons in the *substantia nigra*, unlike non-transgenic controls in which all of these were affected [118].
  Soluble α-syn is able to interact with the DA transporter (DAT) through the NAC domain [119], decreasing the amount of DAT in the PM, to allow for an optimal moderate level of synaptic DA reuptake to be accumulated into vesicles. In the event of α-syn aggregation, a decrease in the level of soluble α-syn results, and leads to the increased PM accumulation of DAT, giving rise to a massive entry of DA into the cell and consequent potential generation of ROS [116]. In accordance, the much more neurotoxic A53T mutant (but not A30P) interacts very weakly with DAT and causes an impairment of vesicular DA storage and release [119].
  Interestingly, the dopaminergic neurotoxin MPTP, which causes a PD-like neurodegeneration in rodents, humans and primates, enters into the cell through the DAT as its ionic metabolite MPP^+^. It then targets the mitochondria, inhibits complex I of the electron transport chain, impairs ATP production, induces a loss of mitochondrial membrane potential allowing the release of cytochrome c and generation of ROS, and additionally, increases α-syn mRNA and protein levels. The A53T mutant can enhance the vulnerability of cells to MPP^+^, while α-syn null-mice are resistant to MPP^+^-induced degeneration [120], signifying a fundamental role of α-syn in drug-mediated neurotoxicity.
2. Interactions with polyanions: different glycosaminoglycans (GAGs) are involved in the formation of amyloid plaques in a variety of neurological disorders [121] and some highly sulphated GAGs (heparin and heparan-sulphate) as well as the proteoglycan agrin are able to bind to α-syn and stimulate its fibrillization *in vitro*. Furthermore, agrin and α-syn co-localize in LBs and LNs [122,123].
3. Interaction with polycations: polycations, such as polyamines, are cellular stabilizers of nucleic acids and membranes, and are essential for growth and differentiation. Interaction with α-syn induced the partial folding of α-syn and, consequently, its oligomerization and fibrillization [124] by binding to the negatively charged C-terminus.
4. Interaction with histones: some animal models of PD are created by administration of the pesticide paraquat to mice where it elevates mouse brain α-syn levels [125]. Additionally, paraquat promoted *in vitro* the overexpression and translocation of α-syn into the cell nucleus, where it can interact with highly basic histones to form complexes that trigger its aggregation, and may reflect aspects of the *in vivo* situation [124].
5. α-Syn--α-syn crosslinking: tissue transglutaminase (tTG) catalyzes covalent crosslinking between reactive Lys and Glu residues [126]. Substrates for tTG include Ap, tau (another hallmark of AD) and the NAC fragment of α-syn, all of them are proteins that undergo aggregation in several neurodegenerative disorders. tTG catalyzes α-syn cross-linking, leading to the formation of high molecular weight aggregates *in vitro* [127], mainly associated with the membrane fraction. Increased levels of tTG have been reported in the *substantia nigra* of PD postmortem brains [128].
6. Interaction with membranes: interaction with synaptic vesicles is one of the biological functions of α-syn. The membrane-bound fraction (about a 15% of total), was shown to have a high aggregation propensity and was able to seed aggregation of the cytosolic form of α-syn [55]. Furthermore, fatty acids and anionic lipids are potent inducers of α-syn fibrillization. *In vitro* association of soluble α-syn with lipid bilayers resulted in the formation of amorphous aggregates and filaments [129].
7. Interaction with other proteins: several proteins have been found to interact with α-syn and some of them were shown to stimulate α-syn aggregation *in vitro,* including tau [130], brain-specific protein p25α [131], MAP-1B [132] and tubulin [133], all of which are components of LBs and/or GCIs, leading to cytoskeleton impairment. The mechanism of interaction remains unknown, but all contain basic motifs, suggesting that interaction could be through ionic bonds. On the other hand, transcriptional factors, such as NF-κB or Elk-1, have been found in LBs [134], suggesting that the sequestration of these factors in the cytosol may compromise the coordination of gene expression in degenerating cells. Similarly, high mobility group B-1 protein (HMGB-1), which is a nuclear DNA-binding protein that facilitates the interaction between DNA and transcriptional factors, has been demonstrated to bind directly with filamentous α-syn *in vitro* and it too is present in LBs [135], potentially disturbing gene expression. α-syn also interacts with other important signalling proteins, epitomized by PKC or ERKs, which can affect cellular viability.
8. Proteins inhibiting α-synuclein aggregation:
  - Chaperones: heat shock proteins (HSPs) are a family of chaperones induced by stress conditions. These proteins suppress protein aggregation and participate in refolding and/or degradation. Hsp70 and Hsp40 are components of LBs and/or GCIs and co-localize with α-syn. Overexpression of HSPs is able to suppress α-syn aggregation *in vitro* [136].
  - β- and γ-synucleins: these proteins share some features with α-syn, but lack others. The α-and β-syn have a conserved *C*-terminus; however β-syn is deficient of 11 aa within the NAC region [137]. On the other hand, γ-syn lacks the Tyr rich C-terminal. All the three behave as typical natively unfolded proteins, but there is a little structural variability. β-syn has properties of a typical random coil, whereas α- and γ-syn are slightly more compact and structured [138]. Both are able to form fibrils, while β-syn is not, when incubated under the same conditions, however even β-syn can be forced to fibrillate in the presence of specific metals (Zn^2+^, Pb^2+^, Cu^2+^), pesticides [139] or by addition of GAGs. Interestingly, the addition of β- or γ-syn in a 1:1 ratio with α-syn increased the time duration of the lag phase and decreased the elongation phase of α-syn fibrillization [138], and was completely inhibited at a 4:1 ratio of an excess of β- or γ-syn over α-syn. This suggests that β- and γ-syn may be regulators of α-syn fibrillization *in vivo* [140], potentially acting as chaperones.
9. Phosphorylation: as discussed above, α-syn undergoes extensive phosphorylation at Ser-129 in synucleinopathies and in ageing brains [141] and it is mostly unphosphorylated under normal conditions [71]. The specific phosphorylation at Ser-129 by CK2 resulted in oligomerization and fibrillization. Furthermore, oxidative stress has been described to enhance α-syn phosphorylation [72].
10. Ubiquitin proteasome system (UPS) malfunction: mutated, misfolded or unassembled proteins are ubiquitinated to be degraded. There is evidence that UPS is impaired in several neurodegenerative diseases, including PD. For example, proteosomal subunits and ubiquitinated proteins have been found in LBs [142]. An inhibitory effect of α-syn aggregates on the hydrolytic activity of the 26S proteosome subunit *in vitro* has been reported [143], and a direct interaction between filaments and the 20S subunit has been shown. Accordingly, in transgenic animals, the inhibition of 20/26 S proteasome in *substantia nigra* led to α-syn accumulation and inclusion body formation, and resulted in a relatively selective degeneration of dopaminergic neurons [144]. Hsp70 expression can attenuate α-syn aggregation toxicity, by binding to α-syn filaments, abrogating its proteasomal inhibitory effect [145].
11. Effect of A30P, A53T and E46K mutations: all these three mutants have been shown to accelerate α-syn oligomerization. While A53T and E46K increase fibril formation more rapidly than wt-α-syn and do not alter lipid-vesicle binding, suggesting that enhanced polymerization induces the disease in patients harbouring these mutations [129]. On the other hand, it has been reported that A30P fibrillates slowly, retarding significantly the formation of mature fibrils [35] and binds poorly to vesicles compared with the wt, maybe hindering axonal transport, leading to accumulation and aggregation, and accelerating the initial oligomerization of α-syn.
12. *C*-terminal truncation: *C*-terminal truncated α-syn can increase α-syn-induced toxicity and aggregation ratio. Additionally, co-expression of full-length α-syn and the *C*-truncated form induced the formation of cytoplasmic inclusions and increased the susceptibility of cells to oxidative stress. This suggests that the *C*-terminus can play a role of an intramolecular chaperone by preventing α-syn from fibrillization [62]. Interstingly, *C*-terminally truncated A53T α-syn has been shown to induce the aggregation of full-length A53T protein faster than its wt counterpart, demonstrating that the mutation increases the accelerating effect that the truncated protein has on the aggregation of full-length α-syn [146].
13. Interaction with metals: postmortem analysis of brain tissues from patients with PD confirm the presence of considerable amounts of metals, such as Fe, Zn and Al, in the *substantia nigra* as well as in LBs when compared with healthy age-matched controls [147,148]. α-syn can interact with several polycations, including Fe^2+^, Al^3+^, Zn^2+^, Cu^2+^, Mg^2+^ and Ca^2+^ [149], through the C-terminal domain, and this binding can catalyze protein aggregation. Additionally, some metals, such as Al [150], can induce a conformational change of α-syn from an unstructured to partially folded β-sheet structure intermediates and, lately, to fibrils [98]. Moreover, there is a shift in the Fe^2+^/Fe^3+^ ratio in favour of Fe^3+^, and a significant increase in the Fe^3+^-binding protein ferritin, together with a decrease in GSH content. Nevertheless, the concentration of metals necessary to induce aggregation is controversial. In general, the required concentration above the physiological values of the metals [151]. Hence, it is probable that metal-induced aggregation is carried out by oxidation of redox metals, rather than specific binding [152].

As mentioned above, oxidation can lead to degeneration of dopaminergic neurons, resulting from the increased level of redox-active metals ions (Cu or Fe) within the *substantia nigra*, which initiates a cascade of events, such as α-syn oligomerization, mitochondrial dysfunction, cytotoxicity, and a rise of cytosolic free Ca, leading to cell death.

### 3.3. Overexpression of α-synuclein

As commented above, duplication or triplication of the *α-syn* gene has been reported in early-onset PD patients, suggesting a dose-dependence association with the disease, and genetic polymorphisms in the *α-syn* gene are linked to idiopathic PD by increases in α-syn concentration [40]. Other studies have revealed that the overexpression of α-syn can induce Fe-dependent aggregation [153].

Under physiological conditions, there is an equilibrium between the natively unfolded and the partially folded conformation; therefore a high α-syn concentration may increase the rate of fibrillization due to an increased total concentration of the partially folded fibrillization-prone conformation [105]. Several studies report an increase in α-syn mRNA levels in brains of patients with PD compared to healthy controls [154]. In fact, overexpression of α-syn can generate α-syn immunopositive inclusions, together with alterations in mitochondria, increases in ROS production [155], lysosomal dysfunction [156] and Golgi fragmentation [157], leading to cell death. Of note, all these effects could be partially attenuated by antioxidants [155].

Accordingly, murine models transfected with recombinant viruses that overexpressed wt- α-syn or the A53T mutated form showed the typical features of PD in humans, including a loss of dopaminergic neurons in *substantia nigra*, α-syn inclusions similar to LBs [158] and a reduction in TH levels. In fact, a correlation between the number of α-syn immunoreactive LBs and α-syn mRNA levels has been found [159].

Another useful murine model to study PD is created by the injection of MPTP, which has been reported to enhance the production of α-syn at both mRNA and protein levels, and induce formation of α-syn-positive inclusions in neurons and abnormal locomotor function [160]. Similarly, administration of the pesticide paraquat to mice caused an upregulation of α-syn and formation of α-syn aggregates. A faster PD-like model has been obtained by using an alternative pesticide, rotenone, which is highly lipophilic and readily crosses plasma membranes to allow rapid brain entry [161]. Within brain, it inhibits mitochondrial complex I and increases oxidative stress by ROS production leading to degeneration of dopaminergic neurons and fibrillar α-syn inclusions [162]. It has been demonstrated that at a nM range concentration, α-syn provides neurons protection against serum deprivation, oxidative stress and excitotoxicity, whereas in the μM range, cytotoxicity results [163]. Thus it is clear that there is a strong correlation between α-syn levels and aggregation-derivated toxicity.

### 3.4. Role of oxidative damage induced by Fe in PD

Both enzymatic and non-enzymatic catabolism of DA are accelerated by the presence of redox elements [164]. In particular, the high concentration of Fe in *substantia nigra* may catalyze the conversion of H_2_O_2_, produced during breakdown of DA, to highly reactive hydroxyl radicals by the Fenton reaction, resulting in oxidative damage [165]. In contrast, the superoxide radical is unreactive, but can serve as reducing agent for oxidized metal ions to produce more hydroxyl radicals from H_2_O_2_, via a cycle known as the Haber-Weiss reaction [166]. Oxidative stress may enhance Fe levels by deattachment from ferritin (see below), from heme proteins like haemoglobin and cytochrome c by peroxides and from iron-sulfur proteins by ONOO^.-^ [117]. Iron also catalyzes the conversion of an excess of DA to neuromelanin, an insoluble black-brown pigment that accumulates in all aged dopaminergic neurons. Neuromelanin sequesters redox ions with high affinity for Fe^3+^; however, when bound to an excess of Fe^3+^, neuromelanin tends to become pro-oxidant, reducing Fe^3+^ to Fe^2+^, which is released from neuromelanin and increase the amount of iron available to react with H_2_O_2_ [167].

In cells, the main route of Fe uptake is through the transferrin receptor (TfR) at the cell surface. Each TfR binds to a Fe-laden transferrin (Tf) molecule and internalizes it via endocytosis. The acidic pH of endosomes causes the dissociation and unloading of Fe from Tf, and is then recycled back to the cell surface. In cytoplasm, most available Fe is sequestered by the iron-storage protein ferritin and a small amount is left free. In the case of Fe deficiency, the levels of TfR in the plasma membrane are increased and ferritin synthesis is downregulated, enhancing the availability of free Fe. When there is too much free Fe, TfR levels are downregulated and ferritin synthesis becomes upregulated.

This homeostasis is regulated at the translational level by two cytoplasmic iron regulatory proteins (IRPs), IRP1 and IRP2 [168; 169], based on their coordinately binding to iron-response elements (IREs) within the 5’-untranslated region (5-UTR) of mRNA ferritin and to the 3-UTR of mRNA TfR. When cellular Fe levels are low, IRPs bind to the ferritin 5-UTR, resulting in a block of ferritin translation and bind to the TfR 3-UTR, stabilizing the mRNA TfR and preventing its endonuclease cleavage [117]. Therefore, there is a decrease in Fe storage, an increase in extracellular import and consequently, an enhancement in cytoplasmic Fe levels. In the presence of excess Fe, IRP2 is degraded [170] and IRP1 inactivated, thereby increasing ferritin levels.

There are several lines of evidence that support the contention that Fe accumulation in a number of degenerative disorders may be a primary event, rather than a consequence of the disease:

1. Injection of Fe_3_Cl into the *substantia nigra* of rats has been reported to result in a selective decrease of striatal DA, which supports the assumption that Fe initiates dopaminergic neurodegeneration in PD. This decrease was prevented by infusion of the Fe chelator, desferrioxiamine [171].
2. Neurodegeneration by 6-OHDA is selective for catecholamine neurons, and it is rapidly oxidized to yield cytotoxic catecholaminergic semiquinones and quinones with production of H_2_O_2_ and hydroxyl radicals. Iron-deficient rats are resistant to 6-OHDA neurotoxicity, suggesting that Fe could be a trigger [172]. Moreover, 6-OHDA-induced toxicity has been reversed by the Fe chelator, desferal [173]. These studies indicate that an enhancement of Fe concentration in *substantia nigra* may be upstream in neurodegenerative process associated with PD.
3. By using MPTP injection in monkeys, a number of Fe chelators have been shown to attenuate MPTP oxidative toxicity, suggesting that Fe mediates or accentuates MPTP effects [174].
4. Targeted deletion of the gene encoding IRP2 in mice causes a misregulation of Fe metabolism and neurodegeneration, leading to abnormal brain Fe deposition, ataxia, bradykinesia and tremors [175].
5. Mutation in the gene that codifies for a ferritin subunit, resulted in disease similar to PD, termed neuroferritinopathy, which is characterized by Fe deposits, progressive neurodegeneration and axonal cyst formation with neurofilaments, ubiquitin and tau protein at the periphery, all of them components of LBs [176].
6. Polymorphisms in five genes related to Fe homeostasis (*Tf, TfR*, *frataxin, lactoferrin* and haemochromatosis-related protein gene) have been linked to sporadic PD incidence, suggesting that there are variations in proteins involved in Fe metabolism that contribute to PD pathogenesis [177].

Anther link between Fe and oxidative stress comes from the discovery of upregulation of ferritin and downregulation of TfR in response to conditions of heightened oxidative stress, leading to IRP inactivation [178], and inversely, overexpression of ferritin decreased the oxidative levels [179]. However, there are reports where increases of H_2_O_2_ level enhanced the activation of IRPs [180]. In a study, when the Fe concentration was increased, IRP2 activity disminished, as expected, but IRP1 activity decreased in the beginning and later aberrantly enhanced, indicating the presence of complex feedback loops to mitigate Fe-induced oxidative damage [181]. Another study assessed that there was neither increase in ferritin levels under excess of Fe nor alteration in the IRP-IRE system in PD brains, maybe induced by the sequestering of Fe by α-syn [182].

Another example of IRP-IRE mediated regulation is the regulation of mRNA levels of mitochondrial complex I, which is compromised in PD [183]. The mitochondrial electron transport chain is the major source of free radicals *in vivo*, therefore dysfunction of IRP regulation of complex I or IRE-containing tricarboxilic acid cycle enzymes, that provide substrates for the electron transport chain, would impair mitochondrial function and would lead to exacerbated levels of oxidative stress. Also, an IRE motif has been located in the 5-UTR of murine and human erythroid-specific delta-aminolevulinic acid synthase (eALAS) mRNA which encodes the first, and possibly rate limiting, enzyme of the heme biosynthetic pathway [184], and for which translation is controlled by IRPs.

Fe-oxidative stress has also been shown in several studies to promote α-syn aggregation [153], maybe due to alterations in secondary structure leading to partially folded states, more susceptible to oligomerization [98]. Fe has been identified as a component of LBs [185], showing the tight relationship among Fe, oxidative stress, α-syn and PD. Indeed, in accordance with and adding to the described literature, an IRE has recently been discovered within the 5-UTR of the α-syn mRNA [186].

## 4. Protein-binding Control Sequences within the 5-UTR of the Alpha Synuclein Transcript

RNA-protein interactions play a key role in many fundamental biological processes through their effects on RNA splicing (in the case of the α-syn mRNA, see ref [187]), turnover, post-transcriptional processing such as capping or poly(A) addition [188], transport, localization and translation [189]. RNA binding of regulatory proteins can modulate synthesis of multiple proteins or differential expression from one mRNA, not only by alternative splicing (such is the case of α-syn), but also by the choice of a certain translation initiation codon [190]. In mammals, translation repression by sequence-specific RNA-binding proteins through the 5-UTR can be robust [191]. Another important regulatory element is sited at the 3’-end of the mRNA (3-UTR). This is the poly(A) tail, characteristic of all mRNAs, which can upregulate or downregulate translation depending on its length and the binding of certain regulatory proteins [188].

The best studied example of a small structural element within the 5-UTR that affects the translation of eukaryotic mRNAs is a stem-loop of around 30 nt, termed the iron-responsive element (IRE), whose role on Fe homeostasis has already been described. The IRE RNA stem loop is usually located within 50 nt from the 5’ cap site of a given mRNA, and this distance is functionally important, because ribosomal preinitiation complex binds to this region. Iron-regulatory proteins (IRPs) are the modulators of translation of the downstream cistron through their binding to the IRE. Our laboratory recently identified a fully functional IRE within the 5-UTRs of mRNAs implicated in neurodegenerative diseases, such as that of the amyloid-precursor protein (APP), associated with AD [192]. This APP IRE was found to be related to those found within 5-UTRs of the mammalian TfR and ferritin L- and H-chain mRNAs, conferring Fe-dependent regulation. Screening for drugs that interact with the 5-UTR of APP mRNA has led to the discovery of a number of metal chelators that suppress holo APP translation [193,194], and likely represent the mechanism via which specific experimental AD drugs lower amyloid-β peptide levels through lowering APP translation [195,196].

**Figure 2.**
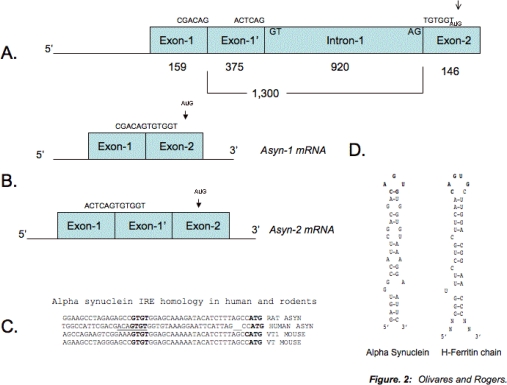
An RNA Stem loop is predicted within 5’ Untranslated region (5’UTR) of the Parkinson’s disease alpha synuclein (α-syn) transcript that is homologous to the Iron-responsive element (IRE) in H-ferritin mRNA. Panel A: The α-syn 5’UTR is encoded by exon-1 and exon-2 of the *α-syn* gene, which can be alternatively spliced to generate either a shorter exon-1/-2 transcript (Panel B upper transcript, [187]), or the alternatively spliced transcript (longer by 375 bases, Panel B lower transcript). Panel B: The alternatively spliced α-syn 5’UTR mRNAs. There is a predominant transcript that encodes a CAGUGU motif at the exon-1/exon-2 splice junction. Also present is the longer alternatively spliced α-syn mRNA variant (Lower transcript) that encodes exon-1 and exon-2 but includes 375 bases of sequences from intron-1. Panel C: Alignment of the α-syn 5’UTR from human, mouse and rat demonstrating the lack of an IRE homology in rodent IREs (in bold is the CAGUGN loop sequences of canonical IREs). Similar to the boxed alignment of the α-syn 5’CAGUGU3’ motif against the IREs of ferritin H- and L- chains (iron storage), ferroportin (iron transport), erythroid eALAS (heme synthesis) mRNAs [186]. Panel D: This α-syn 5’UTR stem loop (ΔG =53 kcal/mol) was predicted by the RNA/FOLD computer program. This α-syn stem loop resembles the classical IRE RNA stem loop (5’CAGUGN3’ loop motif) that controls iron-dependent L- & H-ferritin translation & transferrin receptor (TfR) mRNA stability.

As Figure 2 shows, the recent finding of a putative IRE within the 5’-UTR of α-syn mRNA [186], as encoded by exons 1 and 2, is highly significant. Indeed, two different splicing sites, producing different 5-UTRs have been found by Xia, *et al.* 2001 [187], thus generating one transcript encoding the IRE loop consensus sequence 5’CAGUGU3’ across the splice site junction where, interestingly, the longer transcript encodes the same loop region across its splice junction (Figure 2). The putative α-syn IRE provides a possible mechanism through which Fe can carry out its deleterious action by regulating in some way α-syn expression. In fact, our preliminary data show that the α-syn IRE from the shorter α-syn transcript, indeed, confers desferrioxamine-dependent repression of a luciferase reporter gene in response to iron chelation in SH-SY5Y neuroblastoma cell lines, whereas this sequences has not yet been tested for the longer α-syn alternatively spliced transcript (unpublished data).

Providing more support for a role of α-syn in iron metabolism, a recent finding showed that *α-syn* and the heme metabolism genes erythroid-specific 5-aminolevulinate-synthase gene (*ALAS2)*, ferrochelatase (*FECH)*, and biliverdin-IX beta reductase gene (*BLVRB*) form a block of tightly correlated gene expression co-induced by the transcription factor GATA-1 which is able to noticeably enhance α-syn expression [197]. (Ferrochelatase catalyzes the chelation of iron into protoporphyrin, a precursor of heme group; biliverdin-IX beta reductase gene converts bilirubin from biliverdin, ALAS2 is a key enzyme in heme anabolism). The GATA family of transcription factors, which contain Zn fingers in their DNA binding domains, have emerged as candidate regulators of gene expression in hematopoietic cells. GATA-1 is a hemopoietic transcription factor that specifically occupies a conserved region within α*-syn* intron 1, where several polymorphisms linked to PD have been detected [38]. Endogenous GATA-2 is highly expressed in *substantia nigra* vulnerable to PD, also occupies intron 1, and modulates α*-syn* expression in dopaminergic cells [197].

Interestingly, β- and γ-syn transcripts lack this IRE. This finding suggests that the potential IRP1/-2 binding capacity of α-syn 5-UTR evolved after the divergence of these evolutionally related genes. Interestingly, the human α-syn IRE maintains the CAGUGU loop region motif that is typical of the canonical IRE stem loop, whereas rodents lack it (Figure 2C). This suggests that the capacity of the α-syn IRE to potentially bind IRP1 and/or IRP2 is unique to human α-syn and evolved after the divergence of humans from rodents on the evolutionary timeline.

### 5. Conclusions

In PD, α-syn aggregation is the typical hallmark, and it has been demonstrated that MPTP-induction and α-syn overexpression triggers Fe-mediated α-syn oligomerization, because Fe chelation reduced the toxicity exerted by MPTP. It is known that certain neurotoxins, such as 6-OHDA, are selective for dopaminergic neurons, and cause a PD-like clinical profile and, meaningfully, this neurotoxicity only can be exerted through Fe mediation, since again Fe chelation was able to revert this effect.

The presence of an IRE within the 5-UTR of the α-syn gene and the importance of α-syn in PD, clearly indicates a role for Fe in the pathogenesis of the disorder. Thus, α-syn levels are critical to hold Fe homeostasis, and an impairment in the IRE-mediated regulation system of α-syn can lead to overexpression, to a misfunction in regulation of Fe storage, and consequently to Fe-mediated oxidative stress, α-syn aggregation, dopaminergic neuronal death, DA depletion, and finally to PD symptoms. Indeed, the oxidative insult is not limited to these neurons in *substantia nigra,* and can expand to other areas of the brain leading to massive neuronal death. For this reason, it is frequent to find dementia in patients affected by PD.

Duplication or triplication of the α*-syn* gene leads to α-syn overexpression and aggregation, perhaps by altering the equilibrium between IRE-containing α-syn and IRE-containing Fe regulatory protein system with regard to the recruitment of IRPs, although even a subtle change in α-syn concentration could have the same effect. A shift in α-syn isoform ratio towards the 112 aa isoform, that is more prone to aggregate than the full-length protein, also could occur *in vivo.*

Synucleinopathies are additionally related to AD, since neurofibrillary tangles of protein tau, a hallmark of AD, are in many cases found co-localized with LBs [198]. Moreover, APP mRNA also encodes an IRE, revealing the critical importance of Fe homeostasis in neurodegenerative processes and the main role of the IRE translational regulatory system in the CNS.

In conclusion, the search for new therapeutic agents for PD able to regulate increasing α-syn levels by binding to its IRE might retard Fe-induced neurotoxicity, as well as avoid the deleterious effects of α-syn overproduction and aggregation. Ever since the first-line of treatment of PD, with l-dopa that was developed 50 years ago, there has been no other drug that has proved to be sufficiently efficacious to substitute for it, despite its side effects and short duration of efficacy. Hence, in the future these potential new translation blocker drugs could widen the available clinical options for PD treatment by providing the opportunity for arresting and/or reversing the progression of not only this disease but also other related neurodegenerative synucleinopathies.

## Figures and Tables

**Table 1.** Drugs used to treat Parkinson’s disease.

Class drug	Mechanism of action	Side effects	Specific drug
Anticholinergics	Block acetylcholine receptors	Dry mouth, dry eyes, urinary retention, exacerbation of glaucoma, cognitive impairment	Trihexyphenidy Benztropine Ethopropazine
Amantadine	Blocks NMDA and acetylcholine receptors and promotes release of DA	Cognitive dysfunction, peripheral edema and skin rash	Amantadine
l-dopa	Metabolism to DA in cells containing dopa-decarboxylase	Nausea, hypotension, hallucinations, psychosis, dystonic and choreiform dyskinesias	L-dopa/carbidopa Sinemet CR L-dopa/benserazide
DA agonists	Stimulate DA receptors	Nausea, hypotension, hallucinations, psychosis peripheral edema, pulmonary fibrosis, insomnia	Bromocriptine Pergolide Ropinirole Pramipexole
MAO inhibitors	Block MAO-B receptors to reduce DA metabolism	Nausea, dizziness, sleep disorder and impaired cognition	Selegiline
Catechol O-(COMT) inhibitors	Block peripheral COMT Methyltranferase to improve L-dopa pharmacokinetics	l-dopa related side-effect activity exacerbation, diarrhea, urine discoloration	Entacapone Tolcapone

**Table 2.** Drugs accepted by CINAPS.

Agent	Mechanism	Comments
Caffeine	Adenosine antagonist	KW-6002, a specific A_2A_ receptor antagonist in development
Coenzyme Q10	Antioxidant/mitochondrial stabilizer	Dietary supplement; modest symptomatic benefit based on phase 2 studies
Creatine	Mitochondrial stabilizer	Dietary supplement
Estrogen (17 beta estradiol)	Undetermined	
GM-1 ganglioside	Trophic factor	
GPI-1485	Trophic factor	Neuroimmunophilin ligand
Minocycline	Anti-inflammatory/anti-apoptotic	Antibiotic
Nicotine	Undetermined	
Pramipexole	Antioxidant	Dopamine agonist; clinical neuroimaging data demonstrate a possible disease-modifying effect; exact interpretation and clinical meaning of data remain unclear
Rasagiline	Antioxidant/anti-apoptotic	Selective MAO-B inhibitor; symptomatic benefit in early- and advanced-stage PD based on several phase 3 studies
Ropinirole	Antioxidant	Dopamine agonist; clinical neuroimaging data demonstrate a possible disease-modifying effect; exact interpretation and clinical meaning of data remain unclear
Selegiline	Antioxidant/anti-apoptotic	Selective MAO-B inhibitor; DATATOP study failed to demonstrate neuroprotective benefits
